# Supplementary material for: Wnt Signaling Rescues Amyloid Beta-Induced Gut Stem Cell Loss
Source: Cells. 2022 Jan 14;11(2):281. doi: 10.3390/cells11020281 (PMC8774390; doi:10.3390/cells11020281)
Supplement: Supplementary file 1 [file cells-11-00281-s001.zip › cells-1460617 supplementary for 2nd review/Supplement/Supplemental Figure S1.pdf]

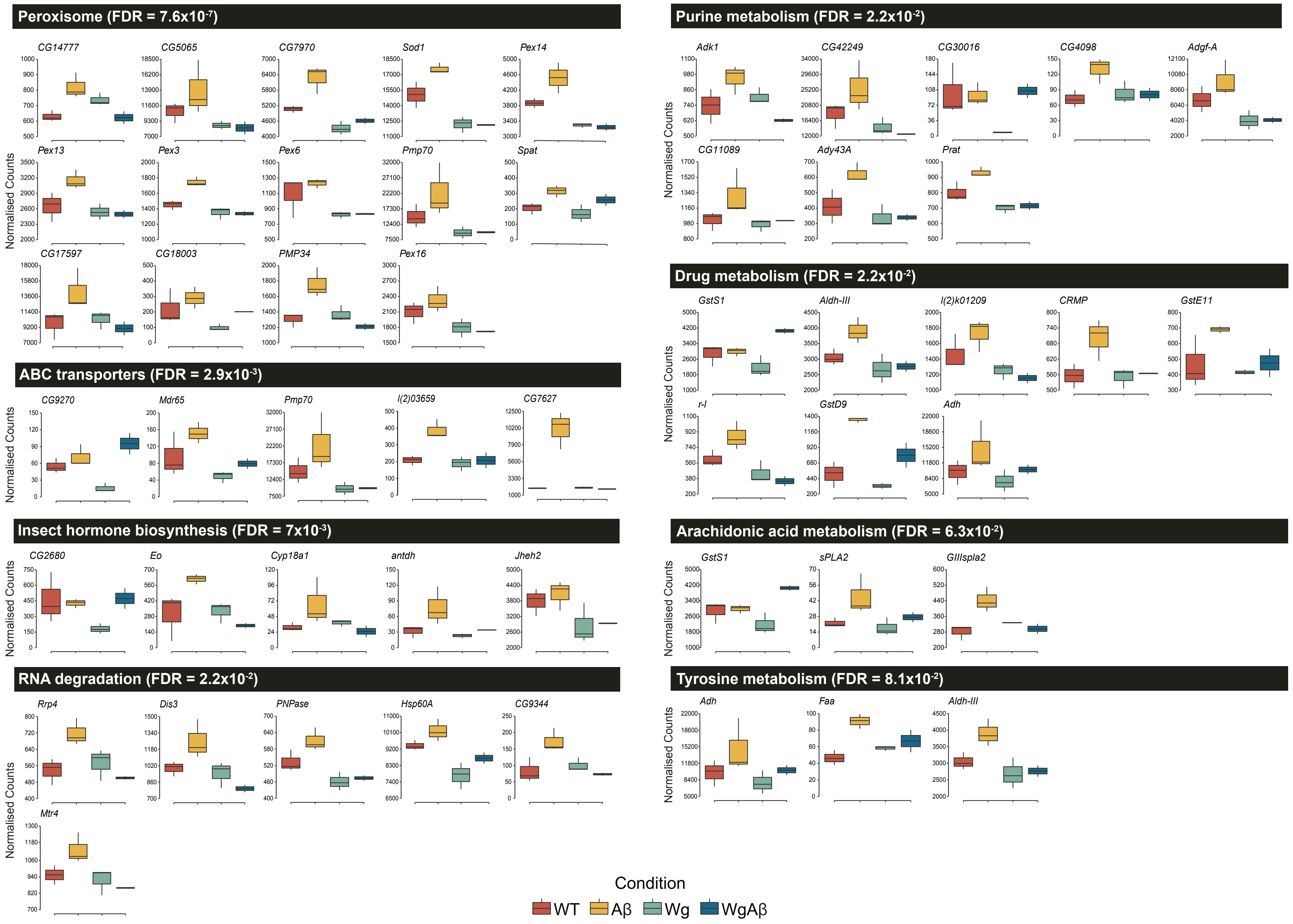
Peroxisome (FDR = 7.6x10⁻⁷)
CG14777
CG5065
CG7970
Sod1
Pex14
Pex13
Pex3
Pex6
Pmp70
Spat
CG17597
CG18003
PMP34
Pex16
Normalised Counts
ABC transporters (FDR = 2.9x10⁻³)
CG9270
Mdr65
Pmp70
l(2)03659
CG7627
Normalised Counts
Insect hormone biosynthesis (FDR = 7x10⁻³)
CG2680
Eo
Cyp18a1
antdh
Jheh2
Normalised Counts
RNA degradation (FDR = 2.2x10⁻²)
Rrp4
Dis3
PNPase
Hsp60A
CG9344
Mtr4
Normalised Counts
Purine metabolism (FDR = 2.2x10⁻²)
Adk1
CG42249
CG30016
CG4098
Adgf-A
CG11089
Ady43A
Prat
Normalised Counts
Drug metabolism (FDR = 2.2x10⁻²)
GstS1
Aldh-III
l(2)k01209
CRMP
GstE11
r-l
GstD9
Adh
Normalised Counts
Arachidonic acid metabolism (FDR = 6.3x10⁻²)
GstS1
sPLA2
GIIIspla2
Normalised Counts
Tyrosine metabolism (FDR = 8.1x10⁻²)
Adh
Faa
Aldh-III
Normalised Counts
Condition
WT
Aβ
Wg
WgAβ

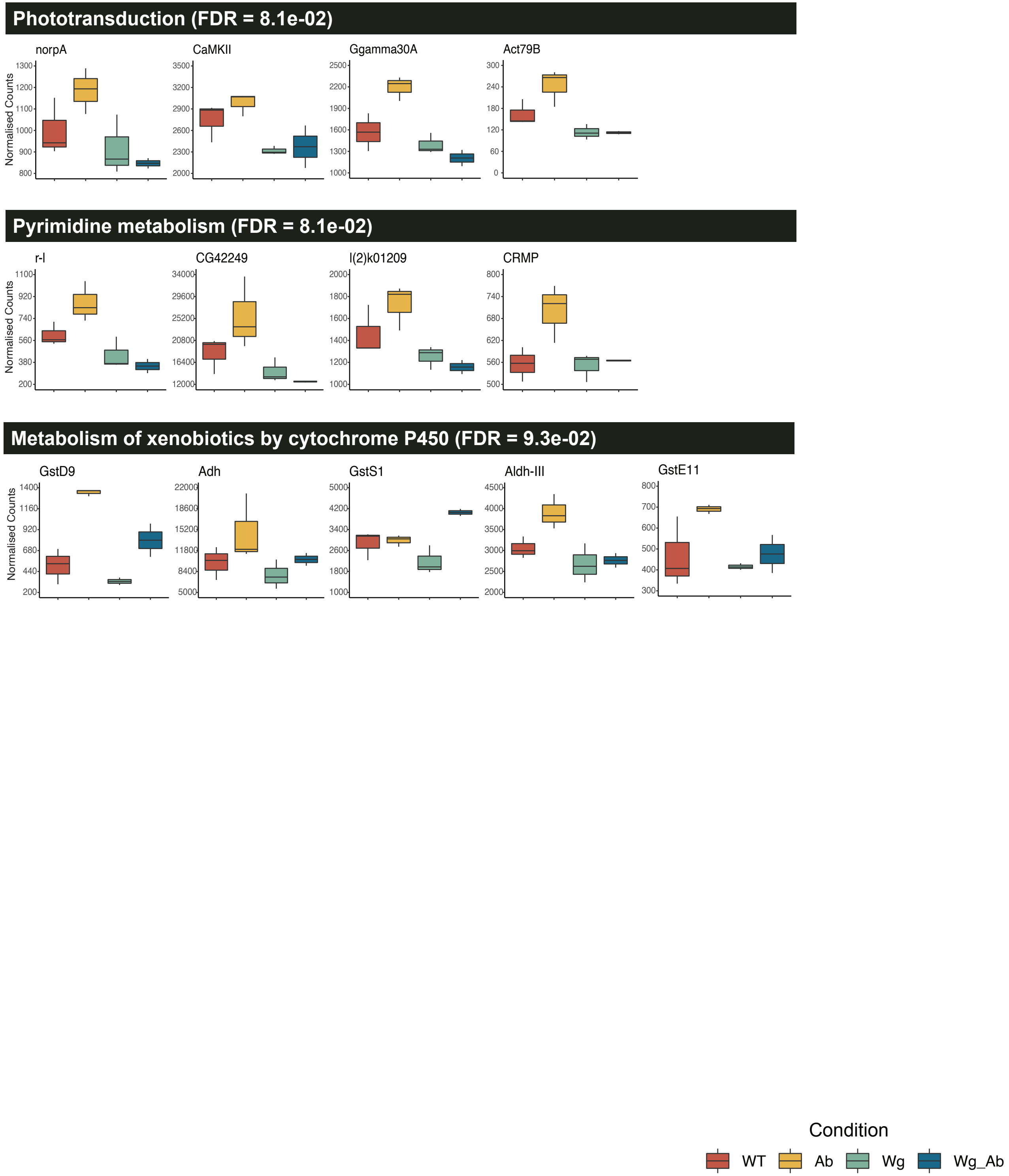
Phototransduction (FDR = 8.1e-02)
norpA
CaMKII
Ggamma30A
Act79B
Pyrimidine metabolism (FDR = 8.1e-02)
r-l
CG42249
l(2)k01209
CRMP
Metabolism of xenobiotics by cytochrome P450 (FDR = 9.3e-02)
GstD9
Adh
GstS1
Aldh-III
GstE11
Normalised Counts
Condition
WT
Ab
Wg
Wg_Ab
