# Supplementary material for: Wnt Signaling Rescues Amyloid Beta-Induced Gut Stem Cell Loss
Source: Cells. 2022 Jan 14;11(2):281. doi: 10.3390/cells11020281 (PMC8774390; doi:10.3390/cells11020281)
Supplement: Supplementary file 1 [file cells-11-00281-s001.zip › cells-1460617 supplementary for 2nd review/Supplement/Supplemental Figure S2.pdf]

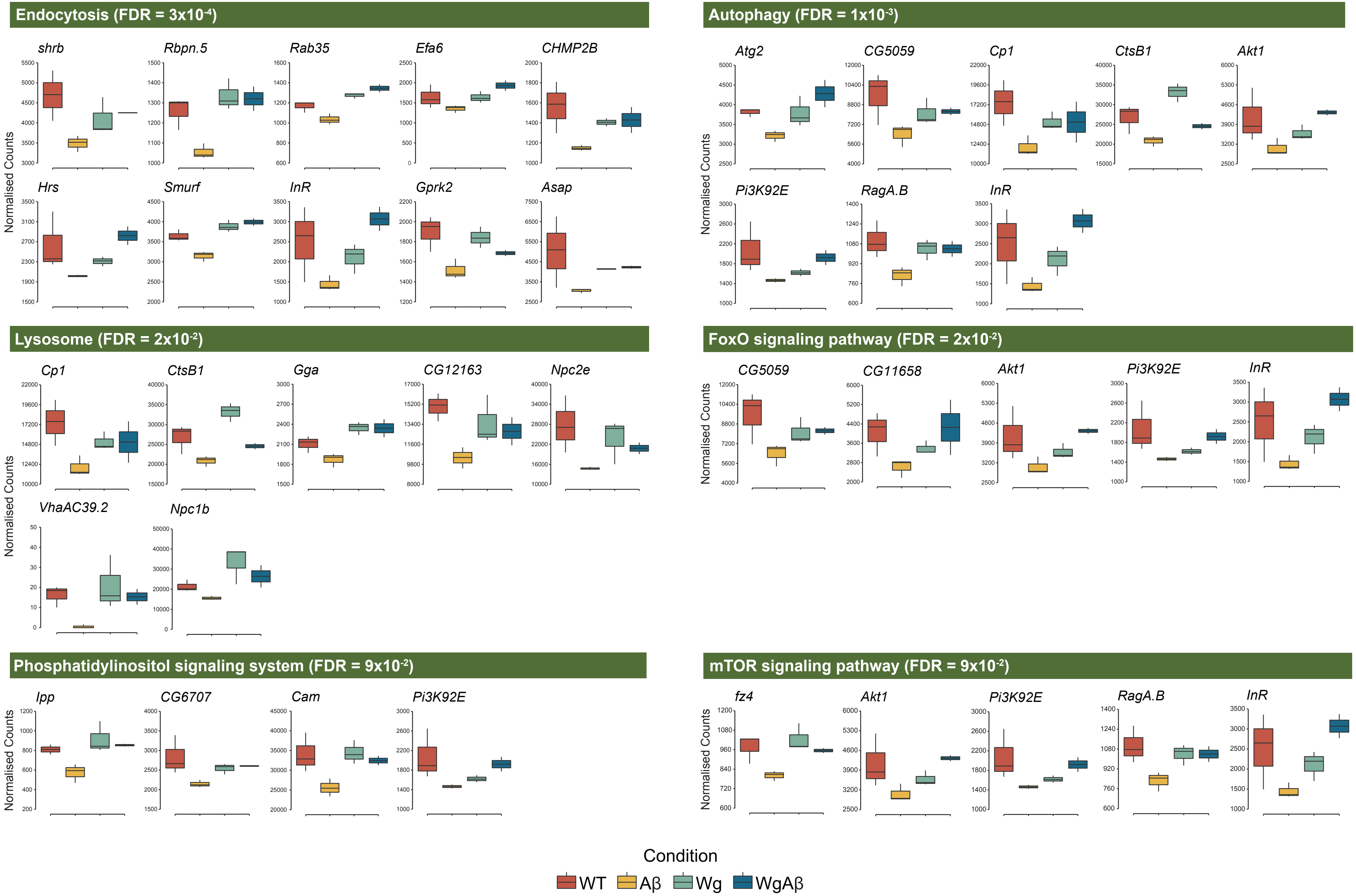

Endocytosis (FDR = 3x10-4)
shrb
Rbpn.5
Rab35
Efa6
CHMP2B
Hrs
Smurf
InR
Gprk2
Asap
Autophagy (FDR = 1x10-3)
Atg2
CG5059
Cp1
CtsB1
Akt1
Pi3K92E
RagA.B
InR
Lysosome (FDR = 2x10-2)
Cp1
CtsB1
Gga
CG12163
Npc2e
VhaAC39.2
Npc1b
FoxO signaling pathway (FDR = 2x10-2)
CG5059
CG11658
Akt1
Pi3K92E
InR
Phosphatidylinositol signaling system (FDR = 9x10-2)
Ipp
CG6707
Cam
Pi3K92E
mTOR signaling pathway (FDR = 9x10-2)
fz4
Akt1
Pi3K92E
RagA.B
InR
Normalised Counts
Condition
WT
Aβ
Wg
WgAβ
