# Supplementary material for: Wnt Signaling Rescues Amyloid Beta-Induced Gut Stem Cell Loss
Source: Cells. 2022 Jan 14;11(2):281. doi: 10.3390/cells11020281 (PMC8774390; doi:10.3390/cells11020281)
Supplement: Supplementary file 1 [file cells-11-00281-s001.zip › cells-1460617 supplementary for 2nd review/Supplement/Supplementary Materials.pdf]

Supplementary Materials: The following are available online at www.mdpi.com

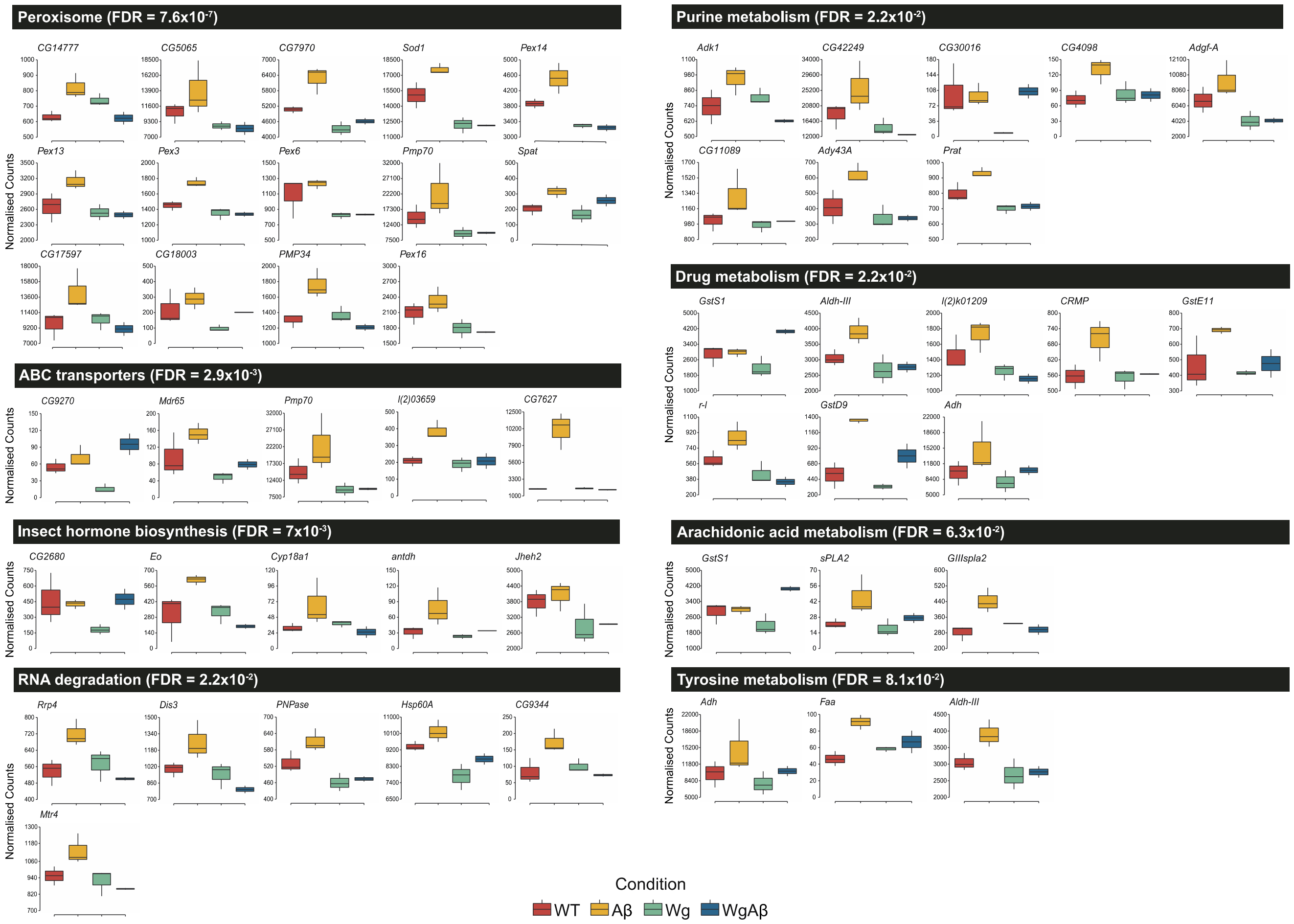

S1: Expression profiles of genes contributing to enrichment of key processes/pathways identified as enriched in Cluster I.

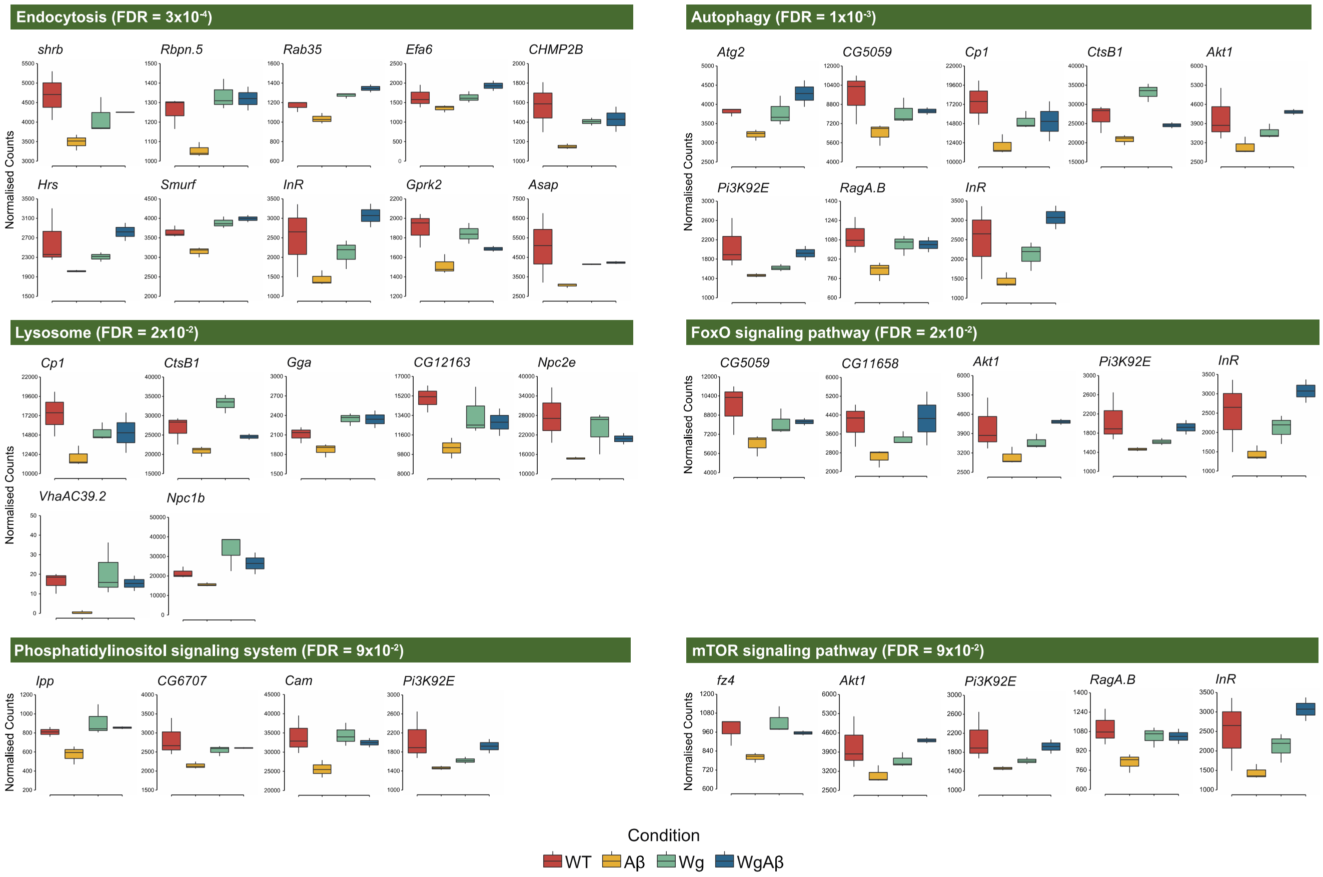

S2: Expression profiles of genes contributing to enrichment of key processes/pathways identified as enriched in Cluster II.

Supplemental Videos

Video 1. Elav-Gal4>UAS-Aβ^{1-42}-CRY2-mCh, Repo-QF2>QUAS-GFP embryos kept in the dark and imaged for neurons using mCherry and glial cells using low (5%) blue laser power to allow imaging of glial cells without inducing pathological clustering. Scale bar represents 50µm.

Video 2. Elav-Gal4>UAS-Aβ^{1-42}-CRY2-mCh, Repo-QF2>QUAS-GFP embryos kept in the light and imaged for neurons using mCherry and glial cells using high (10%) blue laser power to allow imaging of glial cells to induce clustering. Scale bar represents 50µm.

Video 3. Embryos expressing Td-Tomato in the nervous system (Elav-Gal4>UAS-Td-Tomato) develop normally. Scale bar represents 20µm.

Video 4. Expressing wg in the nervous system (Elav-Gal4>UAS-wg, UAS-RFP) is lethal to the embryos. Scale bar represents 20µm.

Supplemental Tables

Table S1: Results from differential expression analysis
Table S2: GO:BP enrichment analysis results for each cluster
Table S3: KEGG pathway enrichment analysis results for each cluster
Table S4: Raw date for Amyloid Lifespans
Table S5: Raw data for Toll Lifespans
Table S6: Raw data for Persephone and Dif Lifespans
